# Evaluating the Effectiveness of Different Irrigant Activation Techniques in Removing the Smear Layer and Opening the Dentinal Canals: A Scanning Electron Microscopic Study

**DOI:** 10.7759/cureus.33961

**Published:** 2023-01-19

**Authors:** Ibrahim Ahmad Ali, Kinda Layous, Hasan Alzoubi

**Affiliations:** 1 Department of Endodontic and Operative Dentistry, Damascus University, Damascus, SYR; 2 Department of Restorative Dentistry and Endodontics, Damascus University, Damascus, SYR; 3 Department of Pediatric Dentistry, Damascus University, Damascus, SYR

**Keywords:** smear layer, scanning electron microscopy analysis, intracanal heating activation, sonic activation, ultrasonic activation

## Abstract

Background

Although irrigation is one of the most important stages during root canal treatment, it is not possible to guarantee the entry of irrigants to all the areas in the root canal, especially in the apical third. Therefore, the activation of irrigants can stimulate and help the irrigants to access hard-to-reach places within the root canal system. Therefore, this study aimed to evaluate the opening of the dentinal canals with scanning electron microscopy (SEM) using different irrigant activation techniques, such as intracanal heating activation, sonic activation, and ultrasonic activation, along the walls of the root canals in the coronal, middle, and apical thirds.

Methodology

The study sample consisted of 36 single-canal premolars, which were randomly divided into the following three equal groups according to the method of activation used: group 1 (n = 12), which involved heating activation inside the root canals, group 2 (n = 12), which involved sonic activation, and group 3 (control; n = 12), which involved ultrasonic activation. Afterward, dental crowns were cut to standardize the working length by 18 mm and were then prepared using the ProTaper Next system up to size X2. Moreover, the irrigant was activated for each group separately, and the teeth were extracted and prepared for SEM. Three images were taken for each sample (i.e., coronal third, middle third, and apical third) at ×2,000 magnification. Data were analyzed using the Mann-Whitney U test.

Results

When studying the removal of the smear layer and the opening of the dentinal canal under ×2,000 magnification, there were no statistically significant differences in the coronal and middle third between the three studied groups. However, statistically significant differences were found in the apical third, where the ultrasonic activation group was the best, followed by the sonic activation group, and the heating activation group.

Conclusions

All methods of activation were effective in removing the smear layer and opening the dentinal canals with the advantage of ultrasonic activation in the rest of the groups. The intracanal heating irrigant activation proved to be similar to the effectiveness of sonic activation and close to ultrasonic activation.

## Introduction

One of the main goals of root canal treatment is to achieve excellent cleaning and optimal sealing of the root canal system to prevent post-treatment bacterial infiltration [1]. Disinfection of the root canal system is a critical factor in the success of endodontic treatment by reducing pathogens [2]. However, root canal preparation alone cannot successfully reduce the bacterial count in the root canal system, and all nickel-titanium files create a smear layer along the root canal walls [3]. Therefore, irrigants are essential for removing debris and disinfecting the root canal system [4].

Sodium hypochlorite solution is one of the most popular and widely used irrigation solutions because it is effectively antibacterial, capable of eliminating and dissolving the remaining and infected dental pulp, lubricating, cheap, and readily available. However, it lacks certain characteristics, such as the ability to remove the smear layer [5]. Therefore, recent literature suggests several techniques to improve the effectiveness of sodium hypochlorite, such as using a large amount of it, activating it in a variety of ways, or preheating it [6]. The most used method of sodium hypochlorite activation is through the use of ultrasonic devices, but this method has several disadvantages, including the inability to reach deep into the curved canals and the possibility of the extrusion of the irrigation solution out of the apex. Although sodium hypochlorite can also be activated through sonic devices, this technique is less effective than ultrasonic activation [7].

Regarding the activation of sodium chlorite solution by heating, Cunningham and Balekjian confirmed that sodium hypochlorite solution allows for faster sterilization at body temperature than at room temperature [8]. Initially, it was suggested to heat sodium hypochlorite before inserting it into the canals, but this was of little effectiveness [9].

Woodmansey demonstrated that sodium hypochlorite solution can dissolve pulp tissue 210 times faster at boiling temperature (90-120°C) than at room temperature [10]. However, this method remained limited due to the lack of studies confirming its safety until Simeone et al. confirmed that when sodium hypochlorite solution is heated inside the canals at a temperature of 150°C for 10 seconds, the temperature of the periodontal tissues does not rise above 42.5°C, which is within the acceptable limits for the periodontal tissue [11].

Considering these advantages of intracanal heating, this study evaluated the activation of sodium hypochlorite solution by heating it inside the root canals using the System B device and compared it with its activation through sonic and ultrasonic devices.

## Materials and methods

Ethical consideration and sample collection

A comparative in vitro study was proposed to compare the effectiveness of the three different sodium hypochlorite solution activation methods (i.e., ultrasonic activation, sonic activation with the EQ-S device, and heating activation within the root canals using the System B device) in removing the smear layer and opening the dentinal root canals of extracted mandibular premolars for orthodontic reasons. The study protocol was approved by the Scientific Research and Postgraduate Board of Damascus University Ethics Committee of Damascus University, Damascus, Syria (IRB number: UDDS-3506-08072019/SRC-527).

The sample size was determined using a sample size calculation program (PS Power and Sample Size Calculation Program, version 3.0.43). The sample size calculation produced a required sample size of 36 mandibular premolars to detect a significant difference (90% power and two-sided 5% significance level).

Sample distribution

Each of the studied teeth was given a number between 1 and 36. Afterward, the teeth were divided randomly through randomization.com into the following three groups: group 1 (n = 12), which involved heating activation inside the root canals, group 2 (n = 12), which involved sonic activation, and group 3 (control; n = 12), which involved ultrasonic activation. Moreover, single-blinded trials were adopted in this study so that the assessors would not know which activation method was used.

Inclusion criteria

The inclusion criteria were mandibular premolars with a single root canal, a straight root canal, intact roots that were not resorbed or infected with caries, teeth that were not previously treated endodontically, and apex size of no more than #30 k-file.

Work procedure

The study was conducted at Damascus University, Faculty of Dentistry, Department of Endodontics, and in the Department of Physics, Laboratory of Atomic Energy, Dobaya. First, the calculus and the attached soft tissues were removed, and the dental radiographs were taken in the buccolingual and mesiodistal directions to ensure that the root canal system was free of any abnormalities and internal resorption. Then, it was cleaned and preserved in 0.9% saline at a freezing temperature (-27°C) according to the preservation protocol of the University of Zurich, Germany [12] until the start of the study to avoid the effect of the preservation solution on the properties of dentin and the dissolution of the pulp tissue.

After completing the sample collection, the crowns were cut using diamond discs and standardized to a working length of 18 mm. Subsequently, the pulp chamber was accessed using a high-speed diamond bur with a water-cooled handpiece, and the orifices of the canals were explored using DG-16 (Hu-Friedy, Chicago, IL, USA). Moreover, the pulp chamber access was refined using Endo-z (Dentsply Maillefer, Ballaigues, Switzerland).

Afterward, a glide path was established using #10 K-file (Dentsply Maillefer), and root canals were prepared using rotary ProTaper Next files (up to X2) (PTU; Dentsply Maillefer, Ballaigues, Switzerland) through an electric motor (VDW, Munich, Germany) at 300 rpm and 2.5 Ncm as well as the irrigation of the canal with 1 mL of saline between each file.

A longitudinal groove was made in the buccolingual direction using discs in preparation for tooth sectioning. After completing the process of irrigant activation, sodium hypochlorite was activated for each group in the following manner.

In the ultrasonic activation group, the root canals were irrigated with 6 mL of 5.25% sodium hypochlorite using a 31-gauge NaviTip (Ultradent Products Inc., South Jordan), which was inserted 2 mm short of the working length. Then, the irrigant ultrasonic activation tips (Dental Satelec Acteon K25, IrriSafe, Satelec Acteon, France; diameter 0.20, taper 0.00, length 25 mm) were inserted 2 mm short of the working length without being in contact with the root canal walls and without being pushed into the canal. Later, the oscillation of the irrigant activation tips was moved 2-3 mm in a coronal-apical direction for 30 seconds, and the power setting was “red 10.” The frequency used under these conditions was 30 kHz. Additionally, the electronic power was measured to be 4 watts, and the displacement amplitude was 65 μm. Afterward, sodium hypochlorite was renewed, and the cycle was repeated thrice.

In the sonic activation group, the root canals were irrigated with 6 mL of 5.25% sodium hypochlorite using a 31-gauge NaviTip (Ultradent Products Inc, South Jordan), which was inserted 2 mm short of the working length. Afterward, the irrigant sonic activation tips (EQ-S; Meta Biomed, Cheongju, Korea; diameter 0.20, taper 0.00, length 25 mm) were inserted 2 mm short of the working length without being in contact with the root canal walls and without being pushed into the canal. Later, the oscillation of the irrigant activation tips was moved 2-3 mm in a coronal-apical direction for 30 seconds, after which sodium hypochlorite was renewed, and the cycle was repeated thrice.

In the intracanal heating activation group, the root canals were irrigated with 6 mL of 5.25% sodium hypochlorite using a 31-gauge NaviTip (Ultradent Products Inc, South Jordan), which was inserted 2 mm short of the working length. Afterward, the head (30.04) of the System B device (VDW, Munich, Germany) was inserted 3 mm short of the working length without being in contact with the root canal walls and without being pushed into the canal. Sodium hypochlorite was activated by being heated until reaching 180°C, moved in a coronal-apical direction for 8 seconds, and then left inactivated for 10 seconds. This process was repeated five times, and the solution was replaced with a fresh solution in each activation cycle.

The activation products were washed with saline and dried with paper points. Then, the samples were immersed in ethanol for 30 seconds and dried with paper points. Moreover, the root orifices were closed with cotton pellets to prevent the entry of debris during sectioning. The sectioning was done longitudinally with a stainless-steel chisel using the grooves made in the beginning. Each sample was divided into thirds by selecting reference points 4 mm and 8 mm from the apex using a scalpel blade.

Afterward, both the mesial and distal halves were examined using the VEGA-II XMU SEM, where several samples were placed within the chamber of the microscope on special holders covered with adhesive. The work was controlled through the accompanying computer by first applying low pressure and then applying an electrical voltage (30 kV) and using ×2,000 magnification.

**Figure 1:**
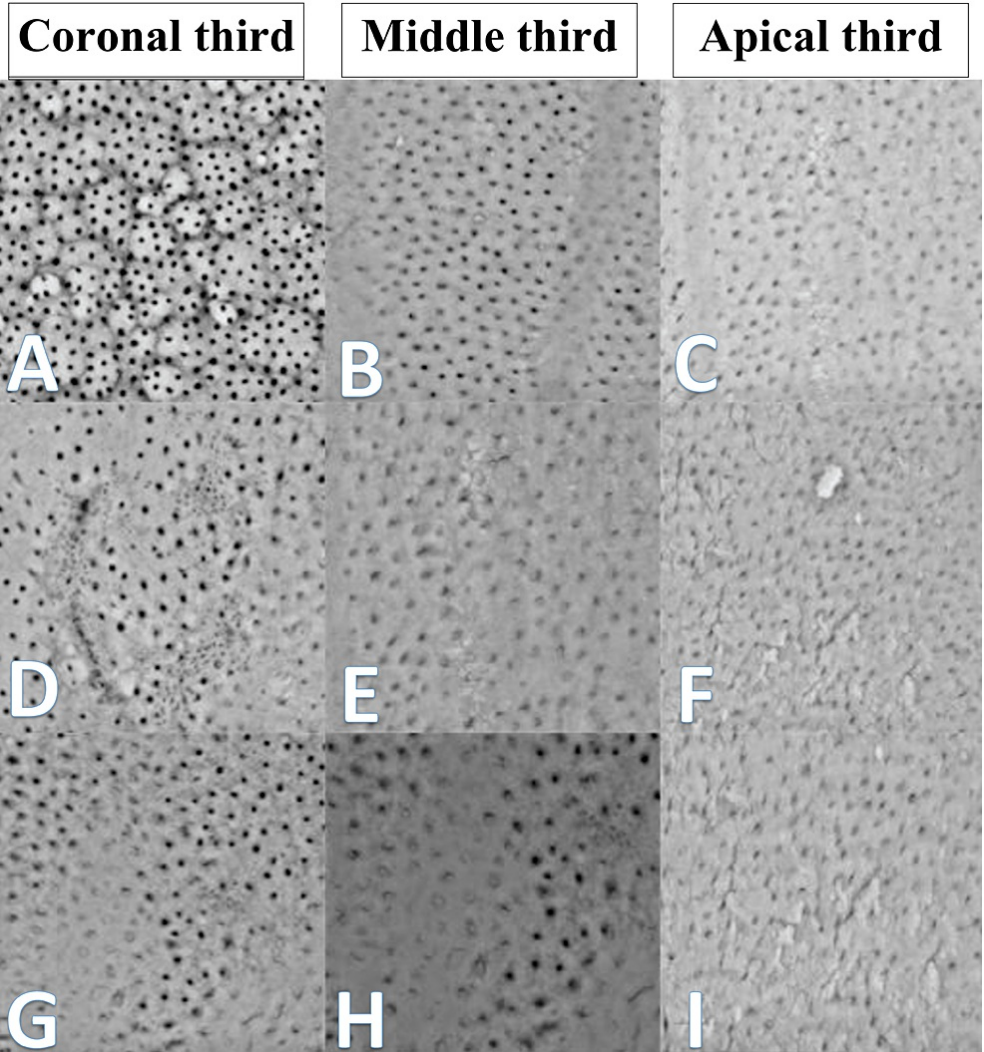
The result of the scanning electron microscope in the coronal, middle, and apical thirds. (A, B, C) ultrasonic activation group, (D, E, F), sonic activation group, and (G, H, I) intracanal heating activation group.

The study adopted the scale developed by Hulsmann et al. to evaluate the smear layer at ×2,000 magnification and observe the opening of the dentin tubules (Figure 1), after which the scores were recorded as follows [13]: score 1 = no smear layer (i.e., open dentinal tubules), score 2 = small amount of smear layer covering the root canal wall (i.e., a few dentinal tubules open), score 3 = homogeneous smear layer covering the root canal wall (i.e., only a few dentinal tubules open), score 4 = complete root canal wall covered by a homogeneous smear layer (i.e., no open dentinal tubule), and score 5 = heavy, nonhomogeneous smear layer covering the complete root canal wall.

Statistical analysis

Statistical analysis was performed using SPSS version 21.0 software (IBM Corp., Armonk, NY, USA). The obtained data were analyzed using the Mann-Whitney U test, and the testing was performed at an α of 0.05.

## Results

The studied sample consisted of 36 extracted mandibular premolars, which were then divided into three groups according to the activation method used (i.e., ultrasonic activation, sonic activation, and intracanal heating activation). To compare and study the differences in the assessment of the smear layer removal between the three thirds within each group, the Kruskal-Wallis test was employed (Tables 1, 2).

**Table 1:** Basic sample characteristics of the smear layer removal between the three thirds within each group.

Groups	Scores	Coronal third	Middle third	Apical third
Ultrasonic activation	Score 1	83.3%	75%	41.7%
	Score 2	16.7%	16.7%	41.7%
	Score 3	0%	8.3%	16.7%
	Score 4	0%	0%	0%
	Score 5	0%	0%	0%
sonic activation	Score 1	66.7%	33.3%	8.3%
	Score 2	33.3%	50%	50%
	Score 3	0%	16.7%	25%
	Score 4	0%	0%	16.7%
	Score 5	0%	0%	0%
Intracanal heating activation	Score 1	58.3%	41.7%	16.7%
	Score 2	41.7%	25%	25%
	Score 3	0%	25%	41.7%
	Score 4	0%	8.3%	8.3%
	Score 5	0%	0%	8.3%

**Table 2:** Kruskal-Wallis test results for the assessment of the smear layer removal in the three thirds within each group. *: statistically significant.

Groups	Thirds	Mean	Test result	P-value
Ultrasonic activation	Coronal third	15.25	5.318	0.070
	Middle third	17.13		
	Apical third	23.13		
Sonic activation	Coronal third	11.83	11.277	0.004^*^
	Middle third	18.42		
	Apical third	25.25		
Intracanal heating activation	Coronal third	12.71	8.216	0.016^*^
	Middle third	18.38		
	Apical third	24.42		

Table 2 demonstrates that there were no statistically significant differences in the smear layer removal between the three thirds in the ultrasonic activation group (p = 0.70), while a statistically significant difference was found in the sonic and intracanal heating activation groups (p = 0.004 and 0.016, respectively). Table 3 presents how the Mann-Whitney U test was applied to compare the three thirds.

**Table 3:** Mann-Whitney U test results for the assessment of the smear layer removal in the three thirds within the sonic and intracanal heating activation groups. *: statistically significant.

Thirds	Groups	Test result	P-value
Sonic activation	Coronal third	-1.803	0.071
	Middle third		
	Coronal third	-3.212	0.001^*^
	Apical third		
	Middle third	-1.808	0.071
	Apical third		
Intracanal heating activation	Coronal third	-1.388	0.165
	Middle third		
	Coronal third	-2.887	0.004^*^
	Apical third		
	Middle third	-1.412	0.158
	Apical third		

Table 3 demonstrates that there was a statistically significant difference in the mean scores of the smear layer removal between the apical and middle thirds in the sonic and intracanal heating activation groups (p = 0.001 and 0.004, respectively). To compare and study the differences in the evaluation of the smear layer removal at ×2,000 magnification between the three groups within each third, the Kruskal-Wallis test was employed (Tables 4, 5).

**Table 4:** Basic sample characteristics of the smear layer removal between the three groups within each third.

Thirds	Scores	Ultrasonic activation	Sonic activation	Intracanal heating activation
Coronal third	Score 1	83.3%	66.7%	58.3%
	Score 2	16.7%	33.3%	41.7%
	Score 3	0%	0%	0%
	Score 4	0%	0%	0%
	Score 5	0%	0%	0%
Middle third	Score 1	75%	33.3%	41.7%
	Score 2	16.7%	50%	25%
	Score 3	8.3%	16.7%	25%
	Score 4	0%	0%	8.3%
	Score 5	0%	0%	0%
Apical third	Score 1	41.7%	8.3%	16.7%
	Score 2	41.7%	50%	25%
	Score 3	16.7%	25%	41.7%
	Score 4	0%	16.7%	8.3%
	Score 5	0%	0%	8.3%

**Table 5:** Kruskal-Wallis test results for the assessment of the smear layer removal in the three groups within each third. *: statistically significant.

Thirds	Groups	Mean	Test result	P-value
Coronal third	Ultrasonic activation	16	1.782	0.410
	Sonic activation	19		
	Intracanal heating activation	20.5		
Middle third	Ultrasonic activation	13.83	4.208	0.122
	Sonic activation	20.58		
	Intracanal heating activation	21.08		
Apical third	Ultrasonic activation	12.92	5.655	0.049^*^
	Sonic activation	20.67		
	Intracanal heating activation	21.92		

Table 5 demonstrates that there were no statistically significant differences in the smear layer removal between the groups in the coronal and middle thirds (p = 0.410 and 0.122, respectively), while a statistically significant difference was found in the apical third (p = 0.049). As Table 6 shows, the Mann-Whitney U test was employed to compare the three groups in the apical third.

**Table 6:** Mann-Whitney U test results for the assessment of the smear layer removal in the three groups within the apical third. *: statistically significant.

Thirds	Groups	Test result	P-value
Apical third	Ultrasonic activation	-2.000	0.045^*^
	Sonic activation		
	Ultrasonic activation	-2.083	0.037^*^
	Intracanal heating activation		
	Sonic activation	-0.394	0.694
	Intracanal heating activation		

Table 6 demonstrates that there was a statistically significant difference in the mean scores of the smear layer removal between the ultrasonic activation and sonic activation groups (p = 0.045) as well as between the ultrasonic activation and intracanal heating activation groups (p = 0.037) in favor of the ultrasonic activation group. On the other hand, there was no statistically significant difference between the sonic activation and intracanal heating activation groups (p = 0.694).

## Discussion

Irrigation in endodontic treatment is one of the most important ways through which we disinfect the root canal system and clean the preparation and tissue residues that harm the fit between the canal filling material as well as the walls of the canal, which affects the success rate of the treatment given that it is about 50% of the inner canal walls and does not touch the preparation files [14].

Previous studies have shown discrepancies in the methods of root canal cleaning and their comparative results have varied due to the appearance of devices and techniques in both the preparation and irrigation of the root canal. Therefore, this study was conducted to compare the different techniques of irrigant activation of the root canal.

The sample consisted of 36 single-canal mandibular first premolars, and the selected teeth were characterized by an apical foramen size of no more than #30 k-file to avoid false results during the evaluation. Moreover, curved premolars were excluded due to the collision of irrigation and activation heads in the curvature region and the inability to continue apically [15].

The lengths of the teeth were standardized in the studied sample to fix the length of the surface being prepared, thereby ensuring greater uniformity in the study sample. Additionally, the crowns were cut to achieve this goal, thereby obtaining a fixed reference point for the working length [12].

For the study, the ProTaper Next system was adopted because it could provide a taper that would allow the irrigation solution the greatest opportunity to remove residues from the root canal. Moreover, an apical expansion of ISO 0.25 was adopted. Then, the canal was irrigated with saline during the preparation to exclude the effect of sodium hypochlorite solution during work and to focus the findings on the effectiveness of the activation technique.

In the ultrasonic activation group, the activation head was inserted 2 mm short of the working length without touching the walls to ensure that it did not affect the inner walls of the canal. Afterward, it was moved 2-3 mm in a coronal-apical direction for 30 seconds. Later, the irrigant solution was renewed thrice to exchange the solution and avoid the deficiency that occurred as a result of volatilization and evaporation [16].

In the intracanal activation group, the head of the System B device (30-04) was inserted 3 mm short of the working length, and the device temperature was 180 °C. Then, it was activated for 8 seconds and deactivated for 10 seconds, and this cycle was repeated five times [17] due to its safety from many aspects. The temperature of the outer surface of the tooth was not raised above 42.5°C, which is less than the temperature that causes tissue damage (47°C) and reduces the evaporation of sodium hypochlorite [11]. Longitudinal tooth sectioning was adopted for the evaluation of the remains because it could give us an idea regarding the entire length of the canal in the three thirds.

Furthermore, the smear layer removal and dentin canal opening were evaluated through SEM, for which several pictures were taken at the three levels and kept for each sample separately until evaluation. The protocol developed by Hulsmann et al. was chosen as the most reliable way to study the dentinal canal opening [13].

The results of the comparisons between the three groups demonstrated that the three activation methods were effective in removing the smear layer and contributed to the opening of the dentin canals. Moreover, the results of the apical third in the ultrasonic activation group were superior to that of the sonic and intracanal heating activation groups. This differed from the findings of the study conducted by Kharouf et al., where ultrasonic activation did not appear to be superior to sonic activation [18].

Additionally, the results of the coronal third in the sonic and intracanal heating activation groups were superior to the results of the apical third. The apical third contained a greater amount of smear layer compared to the coronal third, which showed a greater opening in the dentin canals because the amount of irrigation solution and the effectiveness of the activation head in it was greater than in the coronal third, along with the fact that the replacement of irrigation solution between each cycle of activation was better coronally.

This study agreed with the findings of the study conducted by Kharouf et al. regarding the superiority of the coronal third over the apical in sonic activation, although it differed from it regarding the superiority of the coronal third over the apical third in the ultrasonic activation group, given that there was no difference between the aforementioned thirds [18].

The study also did not find a difference between the thirds of the ultrasonic activation group, which differed from the findings of the study by Koçak et al., where a statistically significant difference was found between the three thirds. This is because the activation time in the previous study was 20 seconds, while it was 30 seconds in the present study [7].

Finally, the findings of this study agreed with those of Iandolo et al., where intracanal heating activation was effective in removing the smear layer and contributed to the opening of the dentin canals [17].

This study was done on extracted premolars, which is the major limitation of the study. This is because of the inability to determine the response of periodontal ligaments and patients due to the raised temperature of the outer surface of the root for each of the irrigant activation techniques.

## Conclusions

Within the limitations of this study, we found that the irrigant activation methods (i.e., ultrasonic, sonic, and intracanal heating) contributed significantly to the removal of a large part of the smear layer and the opening of the dentin canals using only sodium hypochlorite solution without chelating agents. It was found that intracanal heating irrigant activation was similar to sonic activation and close to the effectiveness of ultrasonic activation.
